# Tuning Electron Spin Coherence in Carbon Nanospheres through Defect Engineering

**DOI:** 10.1021/acsnano.5c07050

**Published:** 2025-07-23

**Authors:** Ruslan Yamaletdinov, Jun Zhang, Wafa Afzal, Byron Villis, Polina Topchiian, Simon Ruffell, Oleg V. Yazyev

**Affiliations:** † Institute of Physics, 27218 Ecole Polytechnique Fédérale de Lausanne (EPFL), CH-1015 Lausanne, Switzerland; ‡ Archer Materials Limited (ASX:AXE), Level 2, 477 Pitt Street, Haymarket NSW 2000, Australia

**Keywords:** carbon nanomaterials, hyperfine interaction, spin decoherence, defects, annealing, electron paramagnetic resonance, simulations

## Abstract

This study investigates spin decoherence in large disordered carbon nanosphere (CNS) particles with a focus on predicting and extending the electron spin qubit decoherence time (*T*_2_). We present and experimentally validate a simple analytical model that predicts *T*_2_ and reveal how defects—such as carbon vacancies, hydrogen chemisorption, and substitutional impurities—along with their concentration and distribution, impact *T*_2_. This model offers insights into the interplay among spin density distribution, structural defects, and isotopic composition, demonstrating the role of defect minimization through controlled annealing in enhancing spin coherence. Through comparison with experimental data, we validate our model and demonstrate that spin polarization in the CNS is likely evenly distributed over a characteristic region of approximately 5 nm for *T*_2_ ∼ 200 ns. Based on these findings, we propose a synthetic protocol involving a confined annealing procedure that extends the spin lifetime in the CNS to up to 362 ns. With observed improvements in *T*_2_, our findings provide valuable guidelines for optimizing electron spin coherence time in quantum devices and spintronic applications.

## Graphical abstract

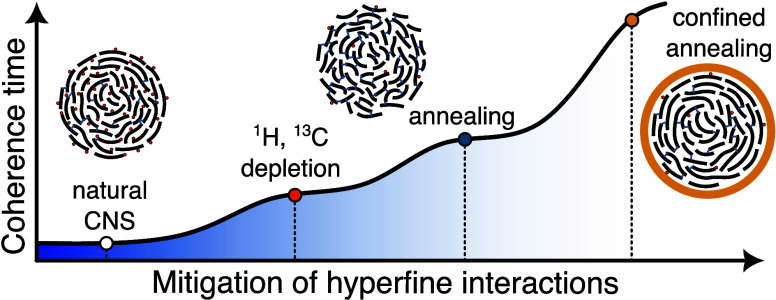

## Introduction

Qubits, or quantum bits, are fundamental units of quantum information. One promising approach for realizing qubits is through well-defined two-level systems that utilize electron spin states.^1,2^ However, the challenge of spin decoherence, along with their high susceptibility to environmental fluctuations, remains one of the major obstacles to practical qubit realization. Generally, two processes limit the lifetime of a qubit state: Relaxation, which transforms the excited state |↑⟩ to the ground state |↓⟩, occurring on the characteristic time scale *T*_1_; and decoherence, which leads to the decay of spin-state superpositions, with a characteristic time scale *T*_2_. In the case of pure dephasing, decoherence may occur even without changing the population of spin states.^3^

One prominent approach to realizing qubits in localized electron states is through III–V semiconductor quantum dots. In these materials, hyperfine interaction (HFI) becomes the major source of central spin decoherence.^4^ Alternatively, carbon- or silicon-based materials, with their small abundance of spin-carrying nuclei ^13^C or ^29^Si and relatively weak spin–orbit coupling, are natural candidates for qubit realization.^5–7^ The spin delocalization and conductivity of graphitic carbon materials also contribute to motional narrowing by eliminating magnetic field fluctuations caused by localized electrons.^8^ A promising realization of long-lifetime spin systems is found in encapsulated bilayer graphene quantum dots^9^ and carbon nanosphere (CNS)—approximately 40 nm spherical soot products composed of 5–10 nm graphitic flakes.^8,10,11^ The electronic properties of these flakes depend on multiple parameters such as size, substituent groups, and edge structure.^11^

The driving interaction for spin relaxation is electron–phonon coupling, which highly depends on the population of low-energy phonons. Consequently, *T*_1_ may drop by several orders of magnitude with increasing temperature from 0 to 300 K.^12^ Changes in environmental conditions caused by electron–phonon interactions may also contribute to the decoherence. However, the reported 50% decrease in *T*_2_ for CNS when cooling from 300 to 2 K^13^ suggests a minimal effect of electron–phonon coupling on the decoherence process. Additionally, the observations of *T*_1_ ≈ *T*_2_ and homogeneous ESR lineshapes in CNS indicate that electron spin–spin dipolar interactions are negligible in this system.^13,14^ Since that, we neglect any electron–phonon interaction and consider nuclei spins as the main source of any environment inhomogeneity, particularly the 1/2 spins of ^1^H, ^13^C, and, in case of substitutions ^14^N, as well as the 3/2 spin of ^11^B.

The Hamiltonian of the interaction of the electron spin with the nuclear spins is

$$\begin{array}{l} {Ĥ}_{hf}={Ĥ}_{iso}+{Ĥ}_{dipolar}=\underset{j}{\sum }A_{j}^{iso}Ŝ{Î}_{j},+\underset{j}{\sum }{Ŝ}^{†}{\mathrm{T̂}}_{j}{Î}_{j}\\ {Â}_{j}^{iso}=\frac{2\mu_{0}}{3}g_{e}\mu_{B}g_{j}\mu_{j}|\psi \left(R_{j}\right){|}^{2}\\ T_{j}^{k,l}=-\frac{\mu_{0}}{4\pi }g_{e}\mu_{B}g_{j}\mu_{j}\left\langle \psi |\frac{3r_{k}^{\prime }r_{l}^{\prime }-\delta_{k,l}r^{\prime 2}}{r^{\prime 5}}|\psi \right\rangle \end{array}$$ 1

where *Ĥ*_iso_—isotropic Fermi contact^15^ (*A* ^iso^ is the isotropic hyperfine interaction constant), and *Ĥ*_dipolar_—dipolar interaction, *g*_e_, μ_B_ are electron *g*-factor and the Bohr magneton, *Ŝ* is the electron spin, *g*_*j*_, μ_*j*_, *Î*_*j*_, and *R*_*j*_ are the magnetic *g*-factor, magneton, spin, and position of the *j*th nucleus, and the sum goes over all of the nuclei, *r*′ = *r* – *R*_*j*_. In the diagonal form of *T̂*_*j*_, *T̂*_*zz*_/2 = −*T*_*xx*_ = −*T*_*yy*_ = *A* ^dip^, where *A* ^dip^ is the dipolar hyperfine interaction constant.

Direct calculation of the HFI requires precise spin density at the nuclei, which can be obtained only through extensive all-electron calculations. However, a simplified approach for calculating HFI with a specific magnetic nucleus can be achieved by considering only the onsite and nearest-neighbor spin densities. Karplus and Fraenkel employed this method to quantitatively describe HFI in sp^2^ carbon atoms through a detailed analysis of exchange interactions.^16^ Using hybrid functional calculations with large all-electron basis sets, Yazyev^17^ further refined and extended this approach by incorporating variations in bond lengths, enhancing the accuracy of the initial model.

Knowledge of the HFI allows for the evaluation of electron spin dynamics in a nuclear spin bath by directly integrating the corresponding master equation.^18^ However, in its general form, this equation must account for all possible configurations of nuclear spins. As the number of nuclei increases, the number of configurations grows extremely fast, making these calculations computationally prohibitive and unreasonably expensive. To simplify this, the spin-bath-induced decoherence can be factorized into irreducible contributions from finite-size bath spin clusters. This factorization is the basis of the cluster-correlation expansion (CCE) method.^19,20^ The order of the CCE is determined by the maximum size of the clusters included in the expansion. While this approach has simplified spin decoherence simulations and expanded their applicability to a wide range of materials,^21,22^ it still relies on precise information about the electron spin density and the positions of bath spins, making it challenging to apply to more complex systems.

This study investigates spin decoherence in metallic CNS particles. We developed a straightforward analytical model to predict decoherence times that aligns well with the results from the CCE simulations. Our model identifies the primary mechanisms contributing to decoherence and highlights the critical structural and isotope distribution factors influencing the quantum state’s lifetime. Through a comparison with experimental data, we validate our model and demonstrate that spin polarization in the CNS is likely evenly distributed over a characteristic region of approximately 5 nm. Based on these findings, we propose a new synthetic protocol involving a confined annealing procedure that extends the spin lifetime in the CNS to up to 362 ns.

## Results and Discussion

CNSs were synthesized through a pyrolysis method as described in previous reports.^10,13^ The materials were collected and subsequently annealed under vacuum conditions before further testing. Adatom-substituted CNSs were prepared by using precursors that contained the desired adatoms. For the control experiments, similar precursors without adatoms were employed. For instance, acetonitrile was used to produce N-doped CNSs, while acetone was used to synthesize undoped CNSs for comparison. The morphological features and internal structure of the synthesized CNS were examined using transmission electron microscopy (TEM), revealing a layered structure with characteristic pattern sizes within ∼10^0^–10^1^ Å (Figures 1a and S5), consistent with previous studies of CNSs.^10,13^ X-ray Photoelectron Spectroscopy (XPS) results indicate a predominantly sp^2^ carbon composition, with residual covalently bonded oxygen content below 10% (Figures 1c and S3). The Raman spectra (Figure 1b) exhibit clear D and G peaks, confirming the graphitic nature of the material and indicating a high density of defects. Unfortunately, the complex interplay of on-site defects,^23,24^ edges, out-of-plane disorder, and interlayer interactions^25^ in CNS do not allow us to use the *I*_D_/*I*_G_ ratio or shift in peak position as a reliable measure of system disorder and precludes a one-to-one mapping between Raman signatures and the spin-relevant defects shown in Figure 1(h–k).^26^ Electron paramagnetic resonance (EPR) motional narrowing (Figure 1d) suggests that these systems have delocalized spin, which aligns with previously observed behavior in similar materials.^8,13^ Experimental observations do not reveal the exact atomistic structure of CNSs, but they suggest the presence of relatively large, interwoven concentric graphitic flakes that form the globular particles of CNSs.^8,10,13^

**Figure 1:**
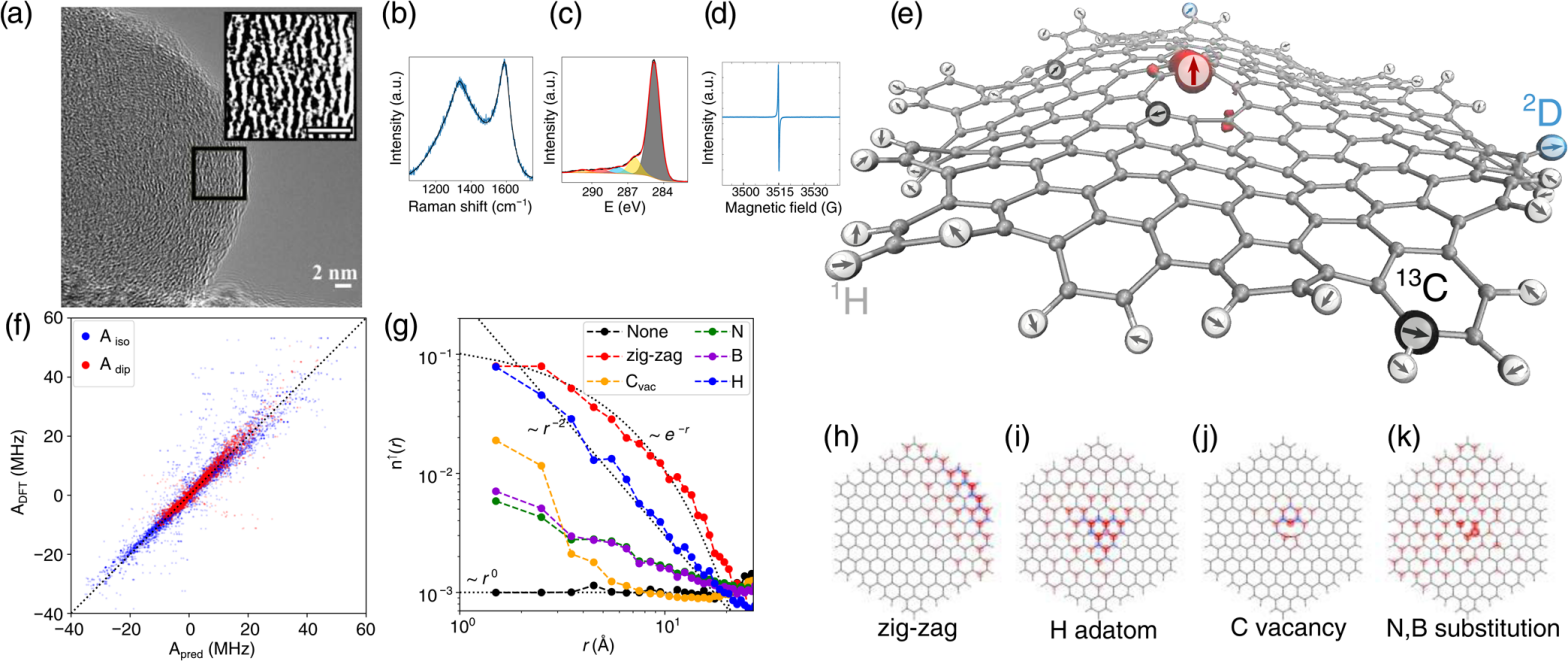
(a) TEM image of a CNS. The inset shows a zoomed-in region with enhanced contrast (scale bar 2 nm). (b–d) Raman, XPS (C 1s), and EPR spectra, respectively, of CNS synthesized using naphthalene precursor. (e) Example of a MD thermalized structure with a carbon vacancy. Red denotes the spin density isosurface, while white, cyan, and black represent randomly distributed ^1^H, ^2^D, and ^13^C nuclei with nonzero spins, respectively. (f) Predicted isotropic (*A*_iso_) and dipolar (*A*_dip_)^13^C HFI versus corresponding *ab initio* DFT values. (g) Sliding average spin density calculated using the GFN2-xTB method (for only centers with *n* > 0 to provide a cleaner picture and to remove spin polarization effects, $n^{↑}\left(r\right)=\frac{1}{N}\sum_{r-w}^{r+w}n_{i}^{↑}$ , *w* = 1.5 Å) as a function of distance from the defect. Dotted lines are guides for the analytical solutions for evenly distributed (*n* ^↑^ ∼ *r* ^0^), single defect quasi-localized state (*n* ^↑^ ∼ *r* ^–2^), and localized state on zigzag edge (*n* ^↑^ ∼ exp­(−*r*)), (h–k) typical spin polarization distribution from GFN2-xTB calculations for structures with different defect types.

Our model assumes the most basic structure for simplicity: Graphene flakes with armchair edges terminated by hydrogen. The hexagonal flakes with different sizes (up to 1014 carbon atoms) and defects were chosen as a model system for the study of electron spin coherence. For each flake size and defect type, we took 5 independent geometrically perturbed structures obtained by 300 K molecule dynamics (MD) annealing. An example of a thermally equilibrated structure with a carbon vacancy is shown in Figure 1e.

Since the flake sizes made the direct calculation of the HFI or spin polarization unreasonably expensive, HFI was calculated based on our parametrization of density functional theory (DFT) results using the refinement Karplus–Fraenkel model,^16,17,27^ where the on-atom spin polarization was estimated using the extended tight-binding model (GFN2-xTB^28^) based on Mulliken spin population analysis. Yazyev’s study on HFI in carbon nanoflakes^17^ demonstrated the model’s accuracy in predicting HFI strengths across various graphitic-like structures, showing strong agreement with DFT results. However, in this study, we extended the model to include some atomic species not covered in the original work. Using the same computational approach, we conducted DFT calculations of HFI for 160 carbon nanoflakes featuring various defects and geometries influenced by thermal fluctuations and reparametrized the model to improve its consistency. Figure 1f compares the predicted isotropic (*A*_iso_) and dipolar (*A*_dip_)^13^C HFI values with their corresponding *ab initio* results. The parametrization results indicate that HFI can be accurately predicted in most cases by considering only the on-site and nearest neighbor spin densities. Further details and results are available in Simulation Details section and Supporting Information S1.

The typical spin polarization distributions from our calculations are shown in Figure 1(h–k) and are consistent with previously known patterns. The spin polarization was obtained by GFN2-xTB^28^ method (see Simulation Details section), and follows the bipartite nature of the honeycomb graphitic lattice. For systems without defects, we observed a homogeneous spin distribution. Systems with a hydrogen adatom, representing a local impurity, exhibited a quasi-localized state with an amplitude decaying as ∼*r* ^–2^ (see Figure 1(g,i)) and an inverse participation ratio (IPR) of $P^{-1}=\sum_{i}^{N}|\Psi \left(i\right){|}^{4}∼\log{\left(N\right)}^{-2}$ , where *i* denotes the lattice site.^29^ For systems with nitrogen or boron substitutions, or carbon vacancies, we observed a greater contribution from extended states mixed with the impurity state, attributed to higher particle-hole asymmetry^30,31^ and sublattice asymmetry.^32^ Truly localized states were found at zigzag edges, where the spin density is known to decay exponentially^33^ (Figure 1g,h), resulting in a weaker size dependence for P .

The decoherence time *T*_2_ for a specific flake can be predicted by using CCE simulations. However, the complexity of this numerical method makes it difficult to directly analyze the factors affecting decoherence. A simpler approach that could potentially yield a fully analytical model—and thus enable a more straightforward analysis—is to estimate *T*_2_ based on the dispersion of the magnetic field Δ*B* created by nuclear spins $\left(T_{2}=\frac{\hbar }{g_{e}\mu_{B}\Delta B}\right)$ .^34^

2 $$\Delta B^{2}=\frac{1}{3}{\left\langle {\left(B_{N}\right)}^{2}\right\rangle }_{N}=\frac{1}{3}\sum_{j}I_{j}\left(I_{j}+1\right)\left(A_{\mathrm{iso},j}^{2}+2A_{\mathrm{dip},j}^{2}\right)$$

The amplitude of Δ*B* ^2^ was estimated by averaging over the ensemble of nuclear wave functions (denoted as ⟨···⟩_*N*_ in eq 2) for specific isotope populations (Figure 2a), with subsequent comparison of the obtained results with CCE simulations for the same isotope distribution (Figure 2b).

**Figure 2:**
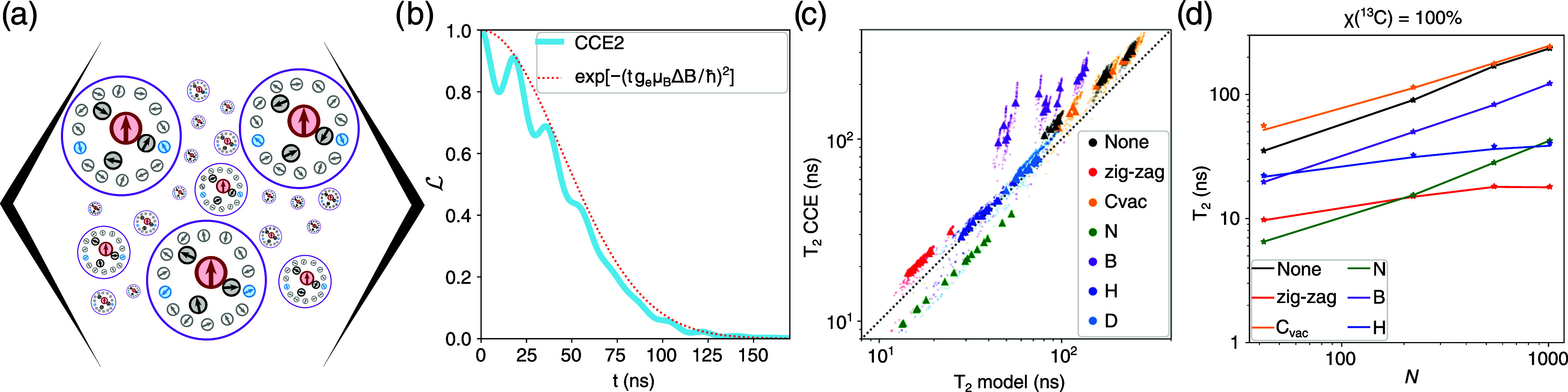
(a) Graphical representation of averaging over the ensemble of nuclei wave functions. (b) Comparison of the coherence function L calculated using second-order CCE with the decoherence according to model eq 2.^34^ (c) Comparison of decoherence time *T*_2_ predicted using second-order CCE versus the analytical model eq 2 for graphene flakes of different sizes, with various defects. Dots represent individual nuclear spin configurations, while triangles denote the average value for each structure. (d) *T*_2_ for different structures, as a function of structure size (*N* is the number of carbon atoms).

Spin decoherence is highly sensitive to the specific distribution of the isotopes. For example, the presence of ^13^C isotopes near a defect has a significant impact on the central electron spin, whereas isotope permutations farther from the defect have a much smaller effect. We assume that a random isotope distribution proportional to their abundance is more realistic. To cover the statistics on isotope distributions, for each structure, we performed 100 separate second-order CCE and Δ*B* ^2^ calculations for random isotope populations. The results of this comparison are presented in Figure 2c. The good agreement between the CCE results and eq 2 highlights the potential of analyzing such a simple equation to derive a straightforward model for *T*_2_. The sum in eq 2 may be divided into sums of averages Δ*B*_*s*_^2^ over chemical species *s* (e.g., C_sp^2^_ or H_edge_), and rewritten into the following form

3 $$\left\langle \Delta B^{2}\right\rangle =\frac{1}{3}\sum_{s}\left\langle \Delta B_{s}^{2}\right\rangle =\frac{1}{3}\sum_{s}\chi_{s}N_{s}I_{s}\left(I_{s}+1\right)\left\langle A_{s}^{2}\right\rangle$$

where summation goes over all chemical specie *s*, *N*_*s*_ is the number of atoms of chemical species *s*, and χ_*s*_ is a fraction of magnetic nuclei (e.g., ^13^C for carbon atoms). It is important to note that in the comparison of *T*_2_ obtained by different methods (Figure 2c), we averaged the *T*_2_ values obtained independently for each distribution of nuclear spins. In most cases, *T*_2_ calculated from ⟨Δ*B* ^2^⟩ does not differ from the one obtained from averaging a set of *T*_2_ values calculated from Δ*B* ^2^, except for the cases of carbon vacancies and hydrogen adatoms, where the difference of $\left\langle {\sqrt{\Delta B^{2}}}^{-1}\right\rangle -{\sqrt{\left\langle \Delta B^{2}\right\rangle }}^{-1}$ for a small χ­(^13^C) becomes prominent. In these cases, there are low probability centers with high HFI, and a more accurate estimation can be obtained by weighted summation of *T*_2_ with and without ^13^C nuclei at the high spin polarization centers. For example, for a carbon vacancy

4 T2=3ℏ[1−χ(C13)]geμB∑s≠Cvac⟨ΔBs2⟩+3ℏχ(C13)geμB∑s⟨ΔBs2⟩

The averaging of the Karplus–Fraenkel expression gives

5 $$\left\langle A_{s}^{2}\right\rangle =\alpha_{s}^{0}\left\langle n_{s}^{2}\right\rangle +\beta_{s}^{0}\left\langle n_{j}n_{k}\right\rangle +\gamma_{s}^{0}\left\langle n_{s}n_{j}\right\rangle$$

where α_*s*_^0^, β_*s*_^0^, and γ_*s*_^0^ can be directly calculated from HFI parametrization (see Supporting Information S1), ⟨···⟩ denotes the average value. Indices *s*, *j*, and *k* correspond to the atom of chemical species *s*, for which the HFI is calculated, and its two nearest neighbors, respectively.

The combination of eqs 3 and 5 gives the dependence of *T*_2_ on the spin density distribution. The values of ⟨*n*_*s*_^2^⟩, ⟨*n*_*j*_ *n*_*k*_⟩, and ⟨*n*_*s*_ *n*_*j*_⟩ required for the calculation of ⟨*A*_*s*_^2^⟩ can be evaluated directly from the *ab initio* simulations for each specific structure. Analytically, *n* is the sum of spin-up and spin-down counterparts, *n* = *n* ^↑^ – *n* ^↓^, where envelope functions for both *n* ^↑^ and *n* ^↓^ are roughly proportional.

Physically, the ⟨*n*_*s*_^2^⟩ can be interpreted as a measure of the degree of localization, or in terms of the inverse (spin) participation ratio, $P^{-1}=\sum_{s}\sum_{i}n_{i,s}^{2}=\sum_{s}P_{s}^{-1}=\sum_{s}N_{s}\left\langle n_{s}^{2}\right\rangle$ . Atoms *j* and *k* are in the same sublattice and *s* and *j* are in different sublattices. The term ⟨*n*_*j*_ *n*_*k*_⟩ indicates the inhomogeneity of the spin density, while ⟨*n*_*s*_ *n*_*j*_⟩ reflects the mutual orientation of the magnetic moments in different sublattices. In the case of slowly varying spin density on carbon atoms in graphene (⟨*n*_*s*_^2^⟩ ≈ ⟨*n*_*j*_ *n*_*k*_⟩ ∼ ⟨n_*s*_ *n*_*j*_⟩), the expected T2∼P/χC13 .

The lowest ⟨*A* ^2^⟩ (upper *T*_2_) limit can be estimated for defect-free structures. In this scenario, the uncompensated spin will be evenly distributed across the structure, leading to ⟨*n*_*s*_^2^⟩ ≈ ⟨*n*_*j*_ *n*_*k*_⟩ ≈ ⟨*n*_*s*_ *n*_*j*_⟩ ≈ *N* ^–2^, and T2∼N/χC13 , which reproduce the known estimation for many systems.^3,35^ However, in our simulations, we observed charge oscillations and edge state polarization even in defect-free structures, likely induced by edge passivation and thermal fluctuations. These effects are well-established in graphene quantum dots.^36,37^ For structures containing defects, a direct evaluation of the overall dependence is more complex. However, considering the envelope function *n*(*r*) ∼ *r* ^–2^, the inverse participation ratio scales as $P∼\log{\left(N\right)}^{2}$ ,^29^ leading to a characteristic decoherence time of *T*_2_ ∼ log­(*N*/χ^13^C).

In systems with multiple nuclear spin species, the amplitude of spin polarization is not the only factor influencing the decoherence. A notable example is a system with a carbon vacancy, where the majority of electron spin density is centered at a single atom. At the natural ^13^C population (χ­(^13^C) = 1.1%), only approximately one out of 90 configurations will have this isotope on an atom with a dangling bond. In other cases, the average spin polarization in the structure is comparable to or even less than that of evenly distributed spin polarization (Figure 1g), which makes the overall dependence of *T*_2_ on *N* similar to that of the defect-free structure (Figure 2d).

Now we turn to the experimental part of our work. *T*_2_ values were measured by using continuous-wave electron paramagnetic resonance (CW-EPR) techniques. For the aromatic hydrocarbon precursor, we measured *T*_2_ ∼ 200 ns, which aligns well with the reported *T*_2_ values for CNS, approximately 175 ns.^13^ The largest considered flake model, containing 1014 carbon atoms, corresponds to a hexagonal structure with a diameter of 11 nm. Figure 2d illustrates the dependence of *T*_2_ on the flake size for different types of defects. The results indicate that at this scale, only defect-free structures and those with carbon vacancies exhibit spin coherence times exceeding 100 ns, with coherence times *T*_2_ ≈ 200 ns for flakes around 5 nm in size. However, earlier mentioned motional narrowing observed in the EPR spectra (Figure 1d) suggests the absence of defects.^8^

From a structural standpoint, the size of the pure graphitic region can be indirectly assessed by the fraction of carbon atoms in the sp^2^ hybridization state (χ­(Csp^2^)). This relationship implies that $T_{2}∼\sqrt{\chi_{{\mathrm{Csp}}^{2}}}$ . By varying the precursor and synthesis conditions, we obtained a set of structures with different χ_Csp^2^_ values, which were confirmed by XPS analysis (Figure 3f). The *T*_2_ measurements exhibit a clear trend of increasing coherence times as the χ­(Csp^2^) fraction increases, and roughly follows the predicted trend (Figure 3f).

**Figure 3:**
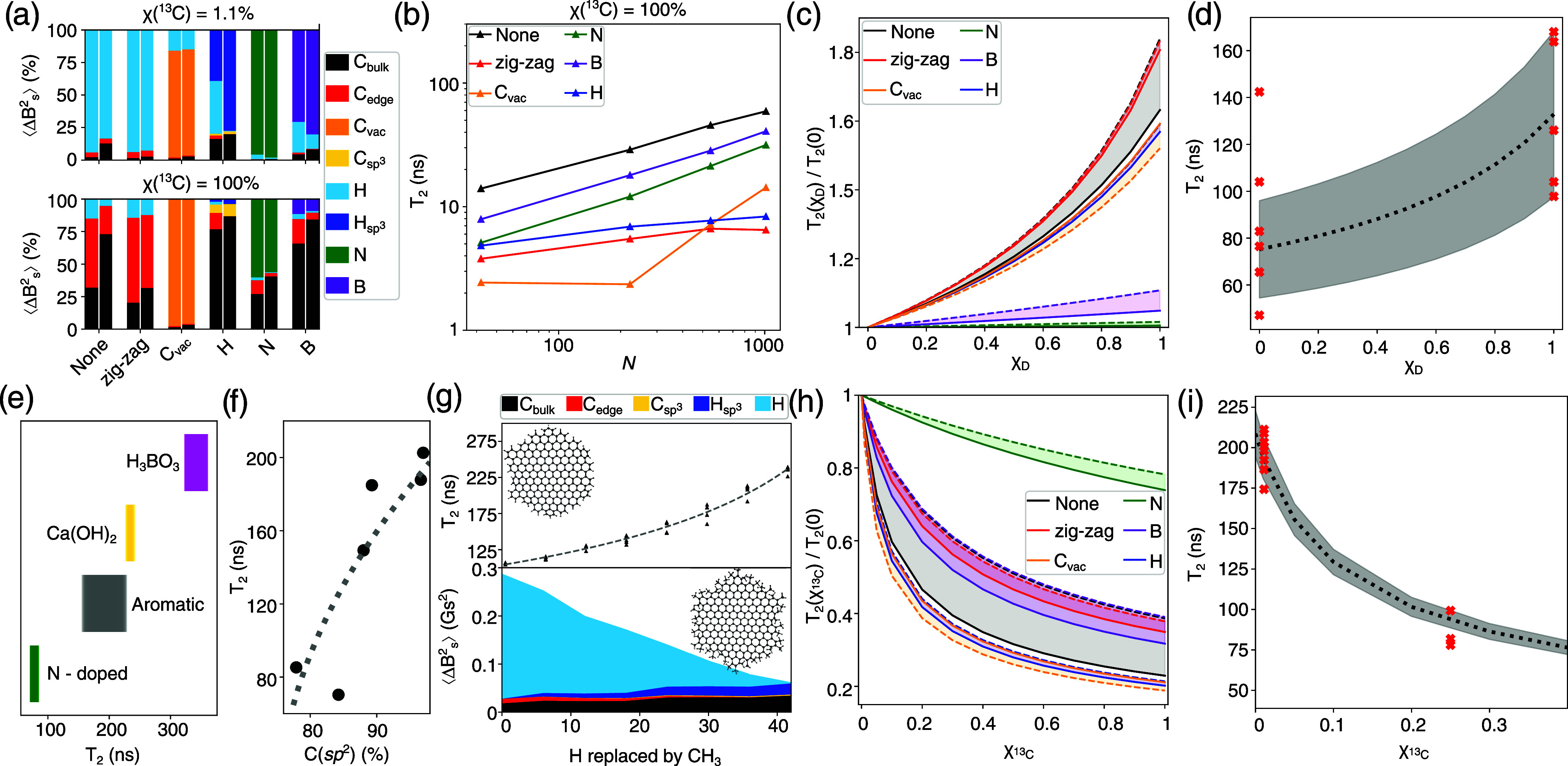
(a) Relative contribution to decoherence of different atomic species in terms of expected dispersion of nuclei’s magnetic field ⟨Δ*B* ^2^⟩. The top panel corresponds to the natural abundance ^13^C (with atomic fraction χ­(^13^C) = 1.1%), and the bottom panel is for χ­(^13^C) = 100%. Two bars for each structure denote the smallest and largest structures (leftmost for *N* = 42 and rightmost for *N* = 1014 carbon atoms). (b) *T*_2_ values for different structures as a function of size (*N* carbon atoms). (e) Experimentally measured decoherence time *T*_2_ for carbon nanospheres synthesized using different precursors and additives (see Figure S3 for more details). (f) Correlation between C sp^2^ content and *T*_2_ for various precursors; the gray dotted line serves as a guide, following the trend $T_{2}∼\sqrt{\chi_{C{\mathrm{sp}}^{2}}}$ . (g) Changes in the decoherence time and ⟨Δ*B* ^2^⟩ upon replacing hydrogen atoms with CH_3_ groups. Inset: Structures of graphene flakes with edges terminated by hydrogen atoms and CH_3_ groups. (c, h) Dependence of the ratio *T*_2_(χ)/*T*_2_(χ = 0) on atomic fraction of the (c) ^2^H and (h) ^13^C isotopes . Solid lines represent the largest structures (*N* = 1014 carbon atoms), while dashed lines correspond to the smallest structures (*N* = 42 carbon atoms). (d) *T*_2_ values for structures synthesized using a natural isotopic composition and 100% deuterated naphthalene at the same conditions. (i) *T*_2_ values for structures synthesized using a natural isotopic composition and 25% ^13^C-enriched xylene. Red symbols denote individual measurements, and dotted lines show model predictions for defect-free structures with flake sizes of 22 and 45 Å for (d, i), respectively. The gray area represents the model uncertainty range (±σ).

To assess the contributions of different electron spin decoherence channels, we calculated the relative ⟨Δ*B*_*s*_^2^⟩ for different model structures (Figure 3a). At the natural isotope abundance and considered flake sizes, hydrogen atoms at the edges play a major role in electron spin decoherence for defect-free structures, primarily due to the low spin density on carbon atoms. Since the structures in our simulations were thermalized, their geometry was perturbed, we observed the additional spin polarization on the edge atoms resulting in enhancing the HFI with hydrogen nuclei. A significant increase (up to 3 times) in decoherence time can be achieved when hydrogen atoms are distanced from (and consequently partially decoupled with) the central spin density by replacing H atoms with CH_3_ (even with an increased total number of ^1^H nuclei) or OH groups (see Figures 3g and S1). However, the substitution of the edge hydrogen atoms with OH or CH_3_ groups without introducing additional in-plane defects can be challenging.

In structures with zigzag edge segments, the spin density near the defect cannot be neglected; however, the earlier discussed likelihood of encountering a ^13^C isotope means that the contribution of carbon atoms to electron spin decoherence is relatively small compared to that of neighboring hydrogen nuclei. This also applies to structures with carbon vacancies.

In doped graphene, the highest spin polarization occurs at the defect atom, making the dopant a significant channel for spin decoherence. For instance, introducing nitrogen through pyrrole or acetonitrile precursors predictably reduces the coherence time, as shown in Figures 3e and S3.

The relatively low contribution to decoherence of the HFI with ^13^C is primarily due to the low natural abundance of this isotope. Increasing χ­(^13^C) to 100% shows a significant increase in contributions of both edge and bulk carbon atoms, with an expected decrease in edge effects as *N* increases (Figure 3a). In structures with point defects, we observe a general trend: A decrease in ⟨Δ*B*_defect_^2^⟩ with increasing χ­(^13^C). This is especially pronounced in the case of hydrogen adatoms, where spin polarization is relatively delocalized, and in the case of boron substitution due to the very weak HFI. The expected *T*_2_ at χ­(^13^C) = 100% (Figure 3b) shows a drastic reduction up to a factor of 30 compared to the natural abundance. In almost all cases, the curves appear scaled-down, except for carbon vacancies, where the decoherence channel (and consequently size dependence) shifts from edge hydrogen atoms to carbon atom with dangling bond. The strongest *T*_2_ decrease is observed for the carbon vacancy case. The overall dependence of the decoherence time on χ­(^13^C) is shown in Figure 3h. To examine this effect experimentally, samples prepared from natural and ^13^C-enriched xylene were prepared. The dependence of *T*_2_ on the ^13^C fraction in the precursor shows a noticeable decrease in decoherence time as χ­(^13^C) increases. This trend aligns well with the theoretical model for defect-free structures (Figure 3i).

Decoherence due to interactions with ^1^H nuclei is relatively significant in many cases; therefore, substituting ^1^H with ^2^H could have a considerable impact on mitigating this decoherence channel, given that the ratio *g*_H_μ_H_/(*g*_D_μ_D_) ≈ 3.25. Indeed, for all nonsubstitution defects, the decoherence time for ^2^H structures is 1.4 to 1.8 times higher (Figure 3c). This effect is most pronounced in cases of evenly distributed spin density and in structures with zigzag defects, and in almost every case, the magnitude of the effect of ^1^H to ^2^H substitution decreases with increasing structure size. The exception is the carbon vacancy, where the predominant mechanism of decoherence involves edge H nuclei and the origin of spin polarization on edges is mainly determined by structural thermal fluctuations. Experimental measurements of fully deuterated samples confirm that the average *T*_2_ is higher compared to nondeuterated counterparts (126 vs 86 ns Figure 3d). However, we want to point out a relatively high dispersion in experimental results (SD = 30 ns). In these experiments, significantly smaller amounts of precursor were used compared with the standard method due to the limited availability of deuterium-substituted precursors. The collected samples were used directly for EPR testing without undergoing high-temperature annealing, which is typically applied to optimize the spin lifetime. Consequently, the spin lifetimes observed in these comparative experiments were shorter than those produced by the standard procedures.

Since experimental evidence, such as values of *T*_2_, motional narrowing, and the dependence of *T*_2_ on isotope content, suggests that the structures are best described by defect-free, we can derive a general relationship for *T*_2_ as a function of the size of confinement region. For a defect-free structure of diameter *d*, the number of carbon atoms $N_{C}=\frac{\pi d^{2}}{3\sqrt{3}a_{0}^{2}}$ (where *a*_0_ = 1.42 Å) and the number of hydrogen atoms $N_{H}=\frac{2\pi d}{3a_{0}}\Theta_{H}$ , where Θ_*H*_ is edge saturation degree. In the ideal case, the spin densities follow the relationships ⟨*n*_*s*_^2^⟩ ≈ ⟨*n*_*j*_ *n*_*k*_⟩ ≈ ⟨*n*_*s*_ *n*_*j*_⟩ ≈ *N* ^–2^. A more accurate approximation for these quantities is *aN*_*C*_^*b*–2^, where *b* is small (Table S5). In this limit, ⟨*A*_*s*_^2^⟩ ≈ *N*_*C*_^–2^ *A*_*s*,0_^2^[1 + *G*_*s*_ log *N*_C_], where *A*_*s*,0_^2^ and *G*_*s*_ can be calculated from the coefficients *a*, *b*, and α_*s*_, β_*s*_, and γ_*s*_ (see Table S6). This approximation aligns well with our previous simulations (Figure 4a) and allows us to find the dependence of *T*_2_ on flake size for large *d*

6 $$T_{2}=\frac{\hbar }{g_{e}\mu_{B}}{\left[\frac{1}{3}\sum_{s}I_{s}\left(I_{s}+1\right)N_{s}N_{C}^{-2}A_{s,0}^{2}\left(1+G_{s}\log N_{C}\right)\right]}^{-1/2}$$

where *N*_*s*_ is the number of atoms of type *s* (e.g., *N*_H_ for hydrogen atoms, χC132πd3a0 for edge carbon atoms).

**Figure 4:**
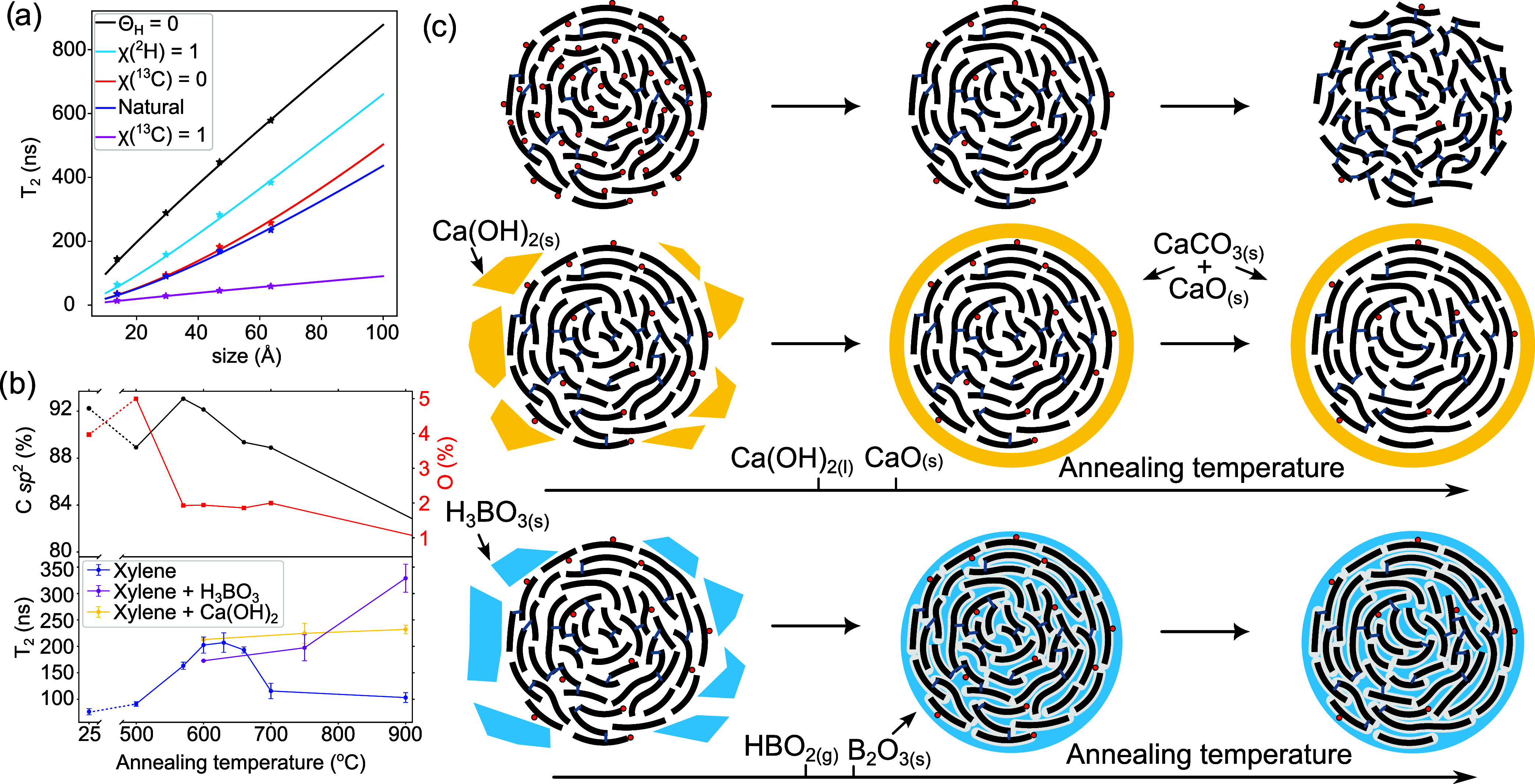
(a) Comparison of the effects of the isotope abundance on decoherence time *T*_2_. Symbols represent the results from flake simulations, while solid lines depict the analytical dependence of *T*_2_ on flake diameter *d*, adapted for a hexagonal flake geometry (eq 6). The legend indicates the factors that differ from the natural state (isotope atomic fractions χ­(^2^H) = 0, χ­(^13^C) = 0.011, and edge saturation degree Θ_H_ = 1). (b) Top panel: XPS results for CNS synthesized from xylene were annealed at different temperatures. Bottom panel: *T*_2_ measurements of CNS annealed without any additives, with the addition of Ca­(OH)_2_, or with H_3_BO_3_. The rising oxygen levels in the early stages are associated with the desorption of polyaromatic hydrocarbon residues. (c) Schematic representation of CNS transformation during annealing. All structural changes are intentionally exaggerated. Top panel (left to right): The initial structure, the structure at the optimal annealing temperature with a decreased concentration of oxygen-containing groups (red circles), and the structure at higher temperatures with increased out-of-plane disorder, smaller graphitic flakes, and an increased number of interlayer *sp* ^3^ carbon cross-links (gray lines). Bottom panel: The effect of annealing with the addition of Ca­(OH)_2_ (yellow) or H_3_BO_3_ (blue), which creates confined conditions and prevents the structure from developing high disorder at elevated temperatures. The ticks on the temperature scale indicate the relative transformation temperatures for Ca­(OH)_2_ and H_3_BO_3_.

In the case of relatively small, fully hydrogenated flakes, where terminal hydrogen atoms are the most important source of decoherence, we expect *T*_2_ ∼ *d* ^3/2^ (Figure 4a, red line) or more precisely

7 $$T_{2}=\frac{\hbar }{3g_{e}\mu_{B}}{\left(\frac{d}{a_{0}}\right)}^{3/2}{\left[\frac{\Theta_{H}}{2\pi }A_{H,0}^{2}\left(1+G_{H}\log\frac{\pi d^{2}}{3\sqrt{3}a_{0}^{2}}\right)\right]}^{-\left(1/2\right)}$$

In the opposite case—where the flake is free of hydrogen and the effect of edge carbons is negligible—*T*_2_ grows sublinearly with flake size (Figure 4a, black line)

8 $$T_{2}=\frac{2\hbar }{g_{e}\mu_{B}}\frac{d}{a_{0}}{\left[\chi_{s}\frac{3\sqrt{3}}{\pi }A_{\mathrm{Cbulk},0}^{2}\left(1+G_{C\mathrm{bulk}}\log\frac{\pi d^{2}}{3\sqrt{3}a_{0}^{2}}\right)\right]}^{-\left(1/2\right)}$$

Neglecting spin density inhomogeneity arising from thermally induced structural perturbations, we recover the known expression for graphene quantum dots T2=2ℏgeμBNCNC13A^3,38^ .

The most straightforward way to increase the decoherence time is by reducing electron spin localization, which can be achieved by lowering the defect density. Inert atmosphere or vacuum annealing, a well-established method for reducing defect concentrations in carbon materials, serve this purpose effectively. Thermogravimetric analysis (TGA) of CNS^10^ reveals typical behavior of graphene-like structures: removal of oxygen-containing groups at *T* ≈ 350 °C and pyrolysis at temperatures above 850 °C. Our XPS results show a pronounced decrease in oxygen-containing groups at *T* > 500 °C, accompanied by an increase in *T*_2_ (Figure 4b), which reaches a maximum at *T* = 630 °C. Further increases in annealing temperature introduce lattice defects,^39^ leading to cracks in graphitic flakes, significant out-of-plane disorder, sp^3^ carbon cross-linking, and the formation of “twisted debris”^40^ (Figure 4c (top)). These structural transformations predictably result in a sharp decrease in the *T*_2_.

Higher-temperature annealing under confined conditions is known to be an effective approach for achieving less defective structures.^40,41^ However, applying pressure to several GPa to a material on a fragile dielectric substrate is challenging. To address this, we created *in situ* confinement conditions by incorporating an internal matrix into the CNS. The approach involves adding relatively inert compounds that would not significantly interact with electron spins, but decompose upon heating to form a solid matrix (Figure 4c (bottom)).

We selected two candidates for such additives: Boric acid (H_3_BO_3_) and calcium hydroxide (Ca­(OH)_2_). Boric acid melts and boils before decomposing at ∼550 °C, while Ca­(OH)_2_ melts and decomposes into solid CaO at ∼600 °C, which partially transforms into CaCO_3_ (see XPS data). Although boron isotopes have nonzero nuclear spins, their HFI are significantly lower than those of ^13^C. Furthermore, no direct evidence of boron incorporation into the graphene lattice was observed (see Supporting Information S3).

Our data does not allow us to conclusively determine the exact structure of the resulting composite. However, we hypothesize that boric acid may distribute more evenly within the CNS, while CaO and CaCO_3_ are less likely to permeate the hydrophobic material. Visual inspection of the annealed products (Figure S4) reveals a wet-like product in samples annealed with H_3_BO_3_, whereas samples treated with Ca­(OH)_2_ are visually indistinguishable from bare CNS.

The results indicate that the addition of Ca­(OH)_2_ enables higher-temperature annealing while maintaining the *T*_2_ value observed for CNS annealed at the optimal temperature (224 ± 8 ns for the xylene precursor at 630 °C). In comparison, the more uniformly distributed boron oxide-based matrix exhibits superior performance at 900 °C, yielding structures with *T*_2_ values reaching up to 362 ns (Figures 3e, 4b, and S3), which corresponds to an increase in the effective spin delocalization length from 6 to 9 nm. This observation is partially supported by the increased flake size seen in samples annealed with H_3_BO_3_ (Figure S5); however, TEM images alone are insufficient to draw conclusions about specific defect types in CNS. These findings highlight the potential of boron oxide (and other potential materials) as an effective additive for enhancing spin coherence in graphitic carbon materials, paving the way to improved performance in spintronic applications.

## Conclusions

To conclude, we developed a straightforward analytical model to predict spin coherence times in carbon nanospheres, incorporating the flake size and disorder parameters. The model accurately captures the interplay among spin density distribution, structural defects, and isotopic composition, providing valuable insights into the dominant mechanisms of spin decoherence. Its predictions align closely with experimental results, particularly in highlighting the effects of the defect concentration and isotope content. Notably, our findings reveal that in metallic carbon nanospheres, spin density tends to distribute relatively evenly within confinement regions.

By identifying key decoherence channels—such as edge hydrogen atoms, carbon vacancy defects, and isotopic variations—this work establishes a robust theoretical framework for optimizing spin coherence in complex carbon-based materials. These insights form the basis for designing advanced quantum materials, emphasizing the critical importance of controlling structural and compositional factors for enhancing coherence times. Addressing these decoherence pathways represents a practical approach to improving the performance of next-generation quantum devices.

Future research should focus on graphitic materials with a precisely controlled isotopic content and defect management. In particular, annealing under confined conditions to minimize defects offers significant potential for achieving longer spin coherence times. These strategies will further the development of carbon materials for quantum technologies, advancing their practical applications in the field.

## Methods and Experimental Section

### Simulation Details

All molecular dynamics (MD) simulations were performed using the LAMMPS package.^42^ The initial geometry preparation was conducted using the AIREBO potential.^43^ To introduce greater structural diversity, small geometry perturbations were induced through annealing at 300 K using the Langevin thermostat for 1 ps with a damping parameter of 0.1 ps. The resulting geometry was then used in subsequent analyses without further relaxation.

The Gaussian16 code^44^ was used for calculating reference spin densities and hyperfine coupling constants. The EPR-III basis set,^45^ specifically designed for predicting hyperfine couplings, was employed in conjunction with the B3LYP hybrid exchange-correlation density functional.^46–48^ We used two different structure sets to further verify the correctness and sufficiency of our fit. The first set replicates the data set used in ref 17, while the second set consisted of randomly generated elliptical hydrogen-terminated graphene flakes with semiaxes *r*_*a*_ ∈ [3; 7] Å and *r*_*b*_ ∈ [4; 6] Å. Each set contained 40 structures for each type of point defect: adsorbed hydrogen, carbon vacancies, boron substitutions, and nitrogen substitutions. To ensure higher structural diversity, all geometries were preliminarily thermalized by using MD.

The HFI parametrization (see Supporting Information S1) was obtained by fitting DFT-calculated hyperfine couplings to the corresponding on-site Mulliken spin densities according to eq S1. We additionally verified that GFN2-xTB provides reasonably good agreement in spin density prediction with a Pearson correlation coefficient of *r* = 0.95.

For CCE vs model comparison, we perform a series of simulations of hexagonal graphitic flakes of 4 sizes (42, 222, 546, and 1014 carbon atoms) with different defects (H adatom, N and B substitution, carbon vacancy, zigzag edge, defect-free). We employ the spin-polarized extended semiempirical tight-binding GFN2-xTB method for accounting for electron–electron interactions.^28^

The coherence function for free induction decay in the 1 μs time scale and 1 ns time step at zero external fields, with $s_{z}=+\frac{1}{2}$ , was calculated using the CCE approach with the PyCCE module.^20^ We verified that the second-order (vs third-order) CCE prediction accuracy does not exceed the accuracy of the stretching exponent fit. The positions of the nuclear spin impurities were chosen randomly according to the natural distribution with a random initial spin-bath state.

### Synthesis and Characterization

CNSs were synthesized through a pyrolysis method as described in previous reports.^10,13^ All chemicals—naphthalene (≥99%), xylene (sulfur-free, 98.5% LR grade), toluene (AR grade), acetonitrile (AR grade), pyrrole (reagent grade, 98%), boric acid (reagent grade, 99.5%), and calcium hydroxide (ACS reagent, ≥95.0%)—were purchased and used without further purification. In a typical synthesis, 1 g of precursor was placed in a crucible bowl and ignited to generate carbon smoke. The resulting carbon material was collected on a glass dish, and any remaining precursor was removed by heating the sample in a vacuum oven at 200 °C overnight. The final powder was then transferred to and stored in a glass sample vial. For postsynthesis annealing treatment, a small portion of as-produced carbon powder was placed in a quartz container inside a tube furnace. The furnace was evacuated to *p* < 10^–4^ mbar before heating and annealing at the desired temperature. Continuous low pressure was maintained with a turbo pump throughout the annealing process. For the confined annealing treatment, CNSs previously annealed at 630 °C were first ground with or without H_3_BO_3_ or Ca­(OH)_2_. The resulting powder was sealed in a quartz tube under vacuum before being placed into a tube furnace for heating and annealing.

The morphological features and internal structure of the synthesized CNS were examined by using transmission electron microscopy (TEM, JEOL JEM-F200) at 200 kV. Raman spectra were recorded by using a Renishaw InVia Raman microscope with a 514 nm laser. X-ray Photoelectron Spectroscopy (XPS, Thermo Scientific K-Alpha) was utilized to examine the surface chemistry and elemental composition of the CNS. Continuous-wave electron paramagnetic resonance (CW-EPR) measurements were conducted by using a Bruker EMX X-band spectrometer to assess the electron spin properties of the CNS at room temperature. The samples were carefully loaded into quartz EPR tubes and sealed under a vacuum to prevent oxidation and environmental interference. Peak-to-peak line width was measured to evaluate spin relaxation properties.

## Supplementary Material



## Notes

Parametrization of hyperfine interactions, and analysis of the effects of structural deformations on coherence times; characterization (XPS, TEM, and Raman) of carbon nanospheres (PDF)
