# Supplementary material for: Tuning Electron Spin Coherence in Carbon Nanospheres through Defect Engineering
Source: ACS Nano. 2025 Jul 23;19(30):27611–9. doi: 10.1021/acsnano.5c07050 (PMC12333433; doi:10.1021/acsnano.5c07050)
Supplement: Supplementary file 1 [file nn5c07050_si_001.pdf]

Supporting Information: Tuning Electron Spin Coherence in Carbon Nanospheres through Defect Engineering

Ruslan Yamaletdinov†, Aaron Zhang, Wafa Afzal‡, Byron Villis‡, Polina Topchiian†, Simon Ruffell‡, and Oleg V. Yazyev†

† Institute of Physics, Ecole Polytechnique Fédérale de Lausanne (EPFL), CH-1015 Lausanne, Switzerland
‡ Archer Materials Limited (ASX:AXE), Level 2, 477 Pitt Street, Haymarket NSW 2000, Australia

S1. HFI PARAMETRIZATION

The combined Karplus–Fraenkel [1] and Yazyev[2] HFI take the form:

$$A_a = \left(S^a + \sum_{j \in NN} Q^a_{a,x_j} + D^a_{a,x_j} \Delta r_{a,j} \right) n_a + \sum_{j \in NN} \left(Q^a_{x_j,a} + D^a_{x_j,a} \Delta r_{a,j} \right) n_j. \tag{S1}$$

where the first and second terms correspond to on-site and nearest neighbors (NN) contributions. n_j is the spin density on atom j, and $\Delta r_{i,j} = |r_j - r_i| - r_0$

The initial approach for hyperfine constant parametrization, proposed by Yazyev [2] (Eq.S1), shows a good linear regression ($R^2 > 0.9$). However, the fit parameters exhibit inconsistency across different datasets, likely due to their relative diversity. Statistical analysis reveals a low correlation between Δr_{ij} (or pyramidization angle θ) and hyperfine parameters for graphene flakes thermalized at 300K (Table S1). The low impact of the geometrical parameters on HFI is explained by relatively minor perturbations of the flakes at the temperature used in our simulations (see SI S2 for details).

TABLE S1. The Pearson correlation coefficient of pyramidization angle (θ), interatomic distance (r) and spin density on atoms (ρ) and HFIs.

	$\tan\theta$	$\tan^2\theta$	r	ρ
A_{iso}	0.008	−0.011	−0.021	0.94
A_{dip}	−0.012	−0.023	−0.052	0.96

Based on these observations, we decided to use the original Karplus–Fraenkel decomposition [1], subsequently neglecting the less significant parameters identified through the smallest impact into the first eigenvector of the covariance matrix. The goal was to create the simplest parametrization, which gave consistent results for different datasets.

The results of this analysis reveal that for sp^2 atoms, isotropic hyperfine interactions (HFIs) are primarily described by the S^a and $Q^a_{\mathrm{C},a}$ terms. We did not find a significant dependence on $Q^a_{\mathrm{H},a}$, likely due to the low spin density on hydrogen atoms. Meanwhile, the only significant parameter for dipolar interaction is S^a. The results of the HFIs parametrization are consistent with those obtained by Yazyev [2], accounting for the differences in the normalization factor (1/2 vs. 1), and are presented in Table S2 and Fig.1(e).

TABLE S2. The results of HFIs parametrization (Eq. S1) (in MHz) for sp^2 hybridized atoms.

	iso			dip	
a	S^a	$Q^a_{\mathrm{C},a}$	R^2	S^a	R^2
^{13}C	86	−24	0.89	67	0.90
^{11}B	13	−27	0.75	28	0.92
^{14}N	63		0.78	230	0.91

The results of the parametrization for the Fermi contact hyperfine parameter for non-sp^2 atoms are presented in Table S3.

TABLE S3. The results of HFI parametrization (Eq. S1) (in MHz) for non-sp^2 hybridized atoms.

a	S^a	$Q^a_{\mathrm{C},a}$	R^2
^{1}H (C sp^3) *iso*	190		0.99
^{1}H (C sp^2) *iso*		−64	0.90
^{1}H (OH) *iso*	1100		0.70
^{13}C (sp^3) *iso*		−38	0.97
^{13}C (vac) *iso*	515	557	0.89
^{13}C (vac) *dip*	35		0.5

The averaging of Karplus-Fraenkel expression gives $\langle A_s^2\rangle$:

$$\begin{aligned}\langle A_s^2\rangle &= \alpha_s^0\langle n_s^2\rangle + \beta_s^0\langle n_j n_k\rangle + \gamma_s^0\langle n_s n_j\rangle;\\ \alpha_s^0 &= S_s^2 + mQ^s_{x_j,s}{}^2; \qquad \beta_s^0 = m(m-1)Q^s_{x_j,s}{}^2;\\ \gamma_s^0 &= 2mS_sQ^s_{x_j,s};\end{aligned} \tag{S2}$$

where S_s and $Q^s_{x_j,s}$ are coefficients described under Eq. S1 [1], m is the number of neighbors. $\langle ...\rangle$ denotes the average value. Indices s, j, and k correspond to the central atom s and two nearest neighbors, respectively. The calculated values of α_s^0, β_s^0, and γ_s^0 are presented in Table S4.

For sp^3 carbon or hydrogen atoms connected to an sp^2 carbon, the on-site density is much smaller than that on the neighboring sp^2 atoms ($n_s \ll n_j$), and the dipolar effect is negligible. In that case,

$$\langle A_s^2\rangle = (Q^s_{x_j,s})^2\left[\sum_j\langle n_j^2\rangle + m(m-1)\langle n_j n_k\rangle\right].$$

When a hydrogen atom is connected to an sp^3 carbon, the HFI is dominantly determined by on-site spin density, and $\langle A^2_{H@sp^3}\rangle = S_s^2\langle n_s^2\rangle$.

TABLE S4. The calculated values of α_s^0, β_s^0, and γ_s^0 ($\times 10^{-3}$ MHz^2) (see Eq. S2, and Table S2).

s	α^0	β^0	γ^0
^{13}C sp^2 bulk	19	3.5	−12
^{13}C sp^2 edge	18	1.2	−8.0
^{11}B bulk	3.9	4.4	−2.1
^{14}N bulk	110		

TABLE S5. sp^2 carbon atoms' spin density quantities fitted as $\sim aN_C^{b-2}$ for bulk and edge carbons in defect-free structures.

	bulk			edge		
	$\langle n_s^2\rangle$	$\langle n_j n_k\rangle$	$\langle n_s n_j\rangle$	$\langle n_s^2\rangle$	$\langle n_j n_k\rangle$	$\langle n_s n_j\rangle$
a	0.66	0.60	0.98	0.79	0.72	0.67
b	0.13	0.09	-0.05	0.24	0.20	0.04

TABLE S6. $\langle A_s^2\rangle$ calculated for a defect-free hexagonal structure as a function of N_C (see Tab S5): $\langle A_s^2\rangle = N^{-2}A^2_{s,0}[1 + G_s \log N_C]$.

s	^{13}C sp^2 bulk	^{13}C sp^2 edge	^{1}H	^{2}H
$A^2_{s,0}\times 10^{-3}$, MHz^2	2.2	9.6	58	5.5
G_s	1.1	0.35	0.24	0.24

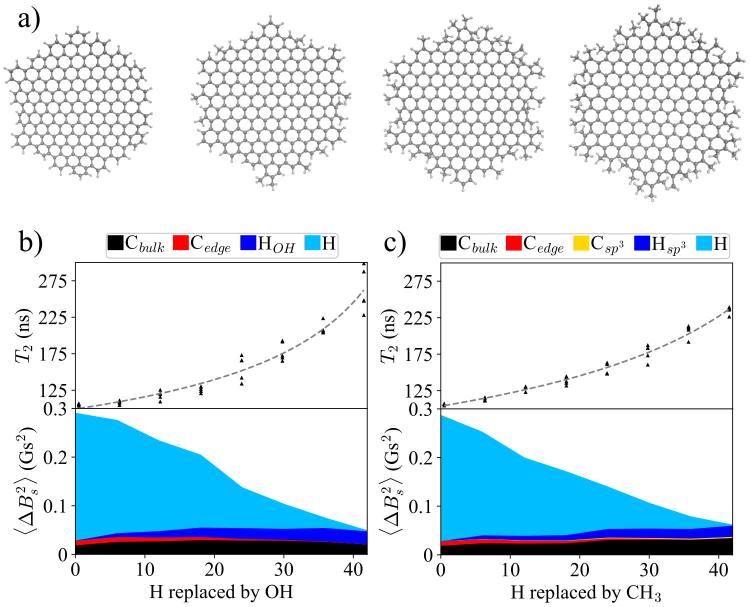

FIG. S1. (a) Structures with hydrogen atoms being replaced by CH_3 groups. From left to right: 0, 12, 30, and 42 out of 42 H atoms replaced. (b) Changes in decoherence time and $\langle \Delta B^2 \rangle$ with hydrogen atoms being replaced by OH groups. (c) Changes in decoherence time and $\langle \Delta B^2 \rangle$ with hydrogen atoms being replaced by CH_3 groups.

S2. EFFECT OF DEFORMATIONS ON T_2

The effect of deformations on decoherence time can be approached from two perspectives: 1) the direct effect of geometrical distortions on the hyperfine parameters (as described by Eq. S1, and due to rehybridization of sp^2 atoms); 2) changes in geometry perturbate the electron wavefunction, leading to spin density redistribution. Since our HFI parametrization does not account for the Δr term, to estimate the amplitude of this effect we used parametrization proposed in [2]

A. Bond length variation

The modification of Eq.5 is

$$\begin{aligned} \alpha_s^r &= ma^2b^2\eta + mc^2\sigma_r^2d^2; \qquad \beta_s^r = mc^2d^2[\eta - \sigma_r^2]; \\ \gamma_s^r &= \gamma_s^0 bd\eta; \quad \eta = \sigma_r^2 + (m-1)\langle \Delta r_{i,j}\Delta r_{i,k}\rangle_s \end{aligned} \tag{S3}$$

where σ_r is the standard deviation of the bond length.

The presence of in-plane deformations, which can be caused by strain or phonons, also contributes to ΔB^2. In the D_{3h} symmetry of the central atom, we can distinguish two types of in-plane deformations: symmetric (A_1'), where $\Delta r_1 = \Delta r_2 = \Delta r_3 = \Delta r$ ($\sigma_r^2 = 0$, $\eta = 2\Delta r^2$); and two uniaxial (E') deformations $\Delta r_1 = -\Delta r_2 = \frac{\sqrt{3}}{\sqrt{2}}\Delta r$, $\Delta r_3 = 0$, and $2\Delta r_1 = -\Delta r_2 = -\Delta r_3 = \frac{1}{\sqrt{2}}\Delta r$ ($\sigma_r^2 = \Delta r^2$, $\eta = 0$ for both cases). The calculated corrections for $\alpha_s^r/\Delta r^2$, $\beta_s^r/\Delta r^2$, and $\gamma_s^r/\Delta r^2$ are presented in Table S7. In the case of uniaxial deformations, the on-site effect cancels out, and the total deformation-induced changes in $\langle A^2 \rangle$ completely vanish for each of the previously considered ideal sublattice spin distributions. The symmetric deformation causes partial suppression of NN effects, but it remains noticeable, especially for (i) ferromagnetic distribution.

B. Rehybridization

The local out-of-plane distortions lead to the rehybridization of p_z orbitals as $p_z \to \sqrt{S}s + \sqrt{P}p_z$, where $S = 2\tan^2\theta$ and $P = 1 - S$ [3, 4], with θ representing the out-of-plane angle. This redistribution of spin density also results in

TABLE S7. The calculated corrections $\alpha_b^r/\Delta r^2$, $\beta_b^r/\Delta r^2$, and $\gamma_b^r/\Delta r^2$ ($\times 10^{-6}$ MHz^2/Å^2), for ideal graphene flake, for symmetric (A_1') and uniaxial (E') deformations.

	$\alpha^r/\Delta r^2$	$\beta^r/\Delta r^2$	$\gamma^r/\Delta r^2$
A_1'	6.32	1.82	6.80
E'	0.911	−0.911	0

changes in the isotropic and dipolar hyperfine coupling constants: $A^{iso} \to PA^{iso} + Sa^{2s}n$ and $A^{dip} \to PA^{dip}$, where $a^{2s} = 3.5 \cdot 10^3$ MHz [2].

The rehybridization-induced changes in A are relatively small and act only on sp^2 hybridized carbon atoms with 3 carbon neighbors. Due to the small bending rigidity compared to the Young modulus of graphene, we can assume that any external stress primarily leads to pure changes in θ, affecting Δr far beyond the small out-of-plane deformation approximation. This assumption implies $\langle\Delta\theta\Delta r\rangle \approx \langle\Delta\theta\rangle\langle\Delta r\rangle = 0$. By neglecting terms with an order of magnitude higher than 2 for small values, we obtain:

$$\begin{aligned}\alpha_b^\theta =& 4\langle\theta^2\rangle_b \left[\delta(2s)a^{2s}a - \alpha_b^0\right],\\ \beta_b^\theta =& -4\langle\theta^2\rangle_b \beta_b^0,\\ \gamma_b^\theta =& 4\langle\theta^2\rangle_b \left[3\delta(2s)ca^{2s} - \gamma_b^0\right],\end{aligned} \tag{S4}$$

where $\delta(2s) = 1$ for isotropic and $\delta(2s) = 0$ for dipolar hyperfine interactions. The calculated values of $\alpha_b^\theta/\langle\theta^2\rangle$, $\beta_b^\theta/\langle\theta^2\rangle$, and $\gamma_b^\theta/\langle\theta^2\rangle$ for ideal graphene flake are $1.54 \cdot 10^6$, $-9.7 \cdot 10^4$, and $-2.11 \cdot 10^6$ MHz^2/rad^2, respectively. The vast majority of the rehybridization effect is associated with the addition of the $2s$ function rather than with reducing the contribution of the p_z orbital, which becomes noticeable in the on-site terms of isotropic interactions. The total magnitude of the effect roughly reproduces the behavior of A^2N^2 for different spin distributions.

C. Effect of temperature on geometry perturbations

To estimate the total effect of geometry perturbations on T_2, we conducted a series of MD simulations at various temperatures to evaluate $\langle\theta^2\rangle$, σ_r^2, η_e, and η_b (Fig. S2). All the values exhibit the expected linear dependence on temperature. The nonzero offset of η is caused by the inequality of C-C bonds for edge and bulk atoms, which depends on the edge structure. The almost constant dependence of η_b might be explained by the compensation of σ_r growth with the decreasing $\langle\Delta r_{i,j}\Delta r_{i,k}\rangle$, attributed to the population of higher energy normal vibrations with increasing temperature.

The obtained values allow us to estimate the temperature effect on T_2 caused purely by lattice perturbations. According to our simulations, for a flat membrane, the decrease in T_2 at 300 K does not exceed 0.3% of the T_2 value at 0 K and can be neglected in most regions except for highly buckled or highly stretched/compressed areas.

S3. CHARACTERIZATION OF CNS

TABLE S8. XPS peak table for CNS synthesised from napthalene.

Name	Peak (eV)	FWHM (eV)	Area (CPS×eV)	At. %
C1s A	284.8	1	64146.3	87.29
C1s B	286.02	0.99	2288.63	3.11
O1s A	532.26	1.28	13001.96	6.33
O1s B	533.78	1.28	647.7	0.32

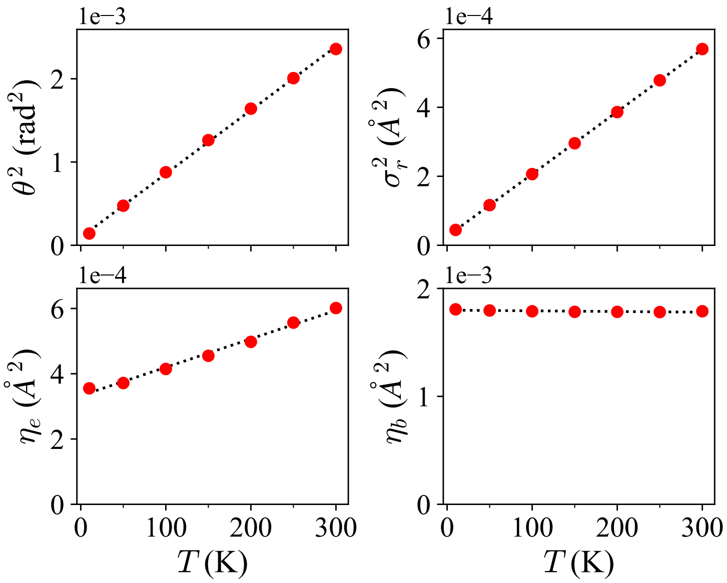

FIG. S2. MD-calculated values of $\langle\theta^2\rangle$, σ_r^2, η_e, and η_b. Dots represent MD results, dashed lines indicate linear fits.

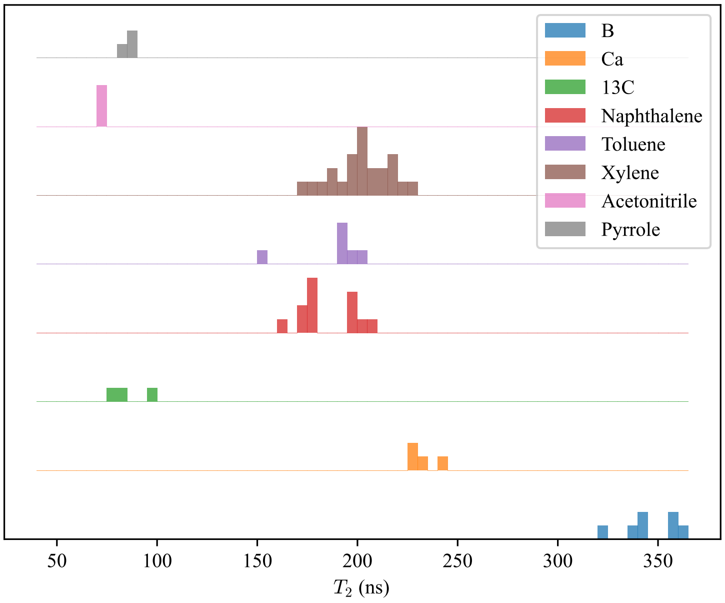

FIG. S3. Measured T_2 values for CNS synthesized from different precursors.

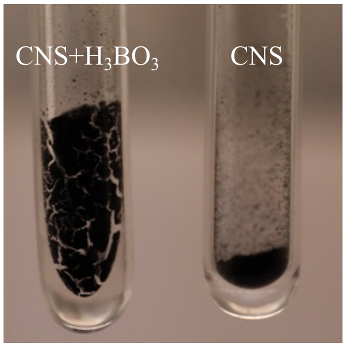

FIG. S4. photo of CNS annealed with and without H_3BO_3 additions.

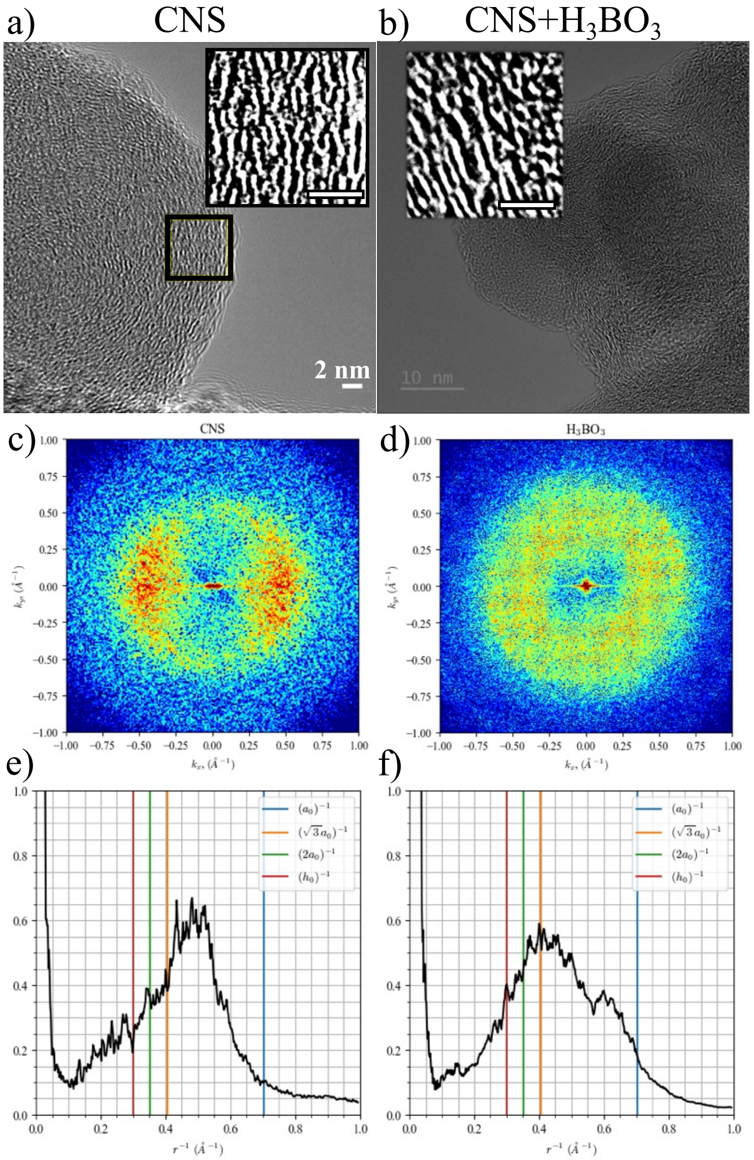

FIG. S5. TEM images of CNS synthesized from xylene with standard annealing procedure (a) and with H_3BO_3 (b). Inset: zoomed image with enhanced contrast (scale bar 2 nm). (c,d): 2D Fourier transform of TEM images. (e,f): angle integrated Fourier transform.

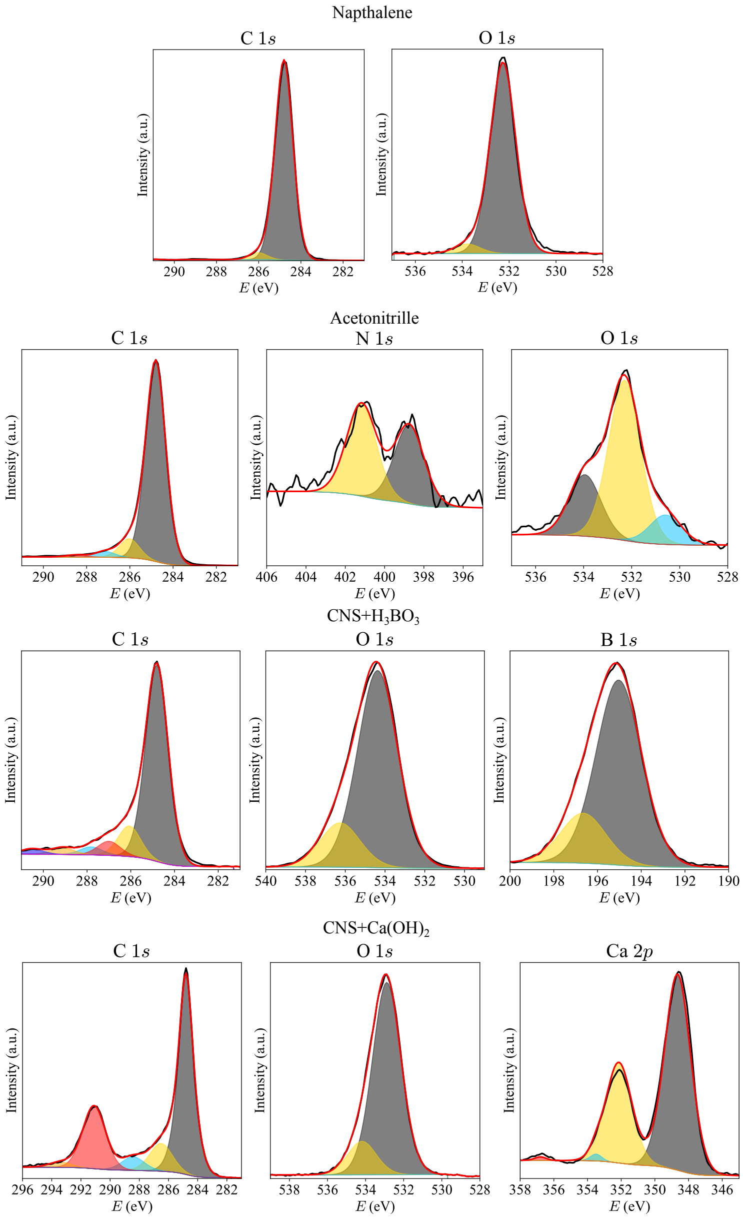

FIG. S6. XPS spectra for CNS synthesized from different precursors.

TABLE S9. XPS peak table for CNS synthesised from acetonitrille.

Name	Peak (eV)	FWHM (eV)	Area (CPS×eV)	At. %
C1s A	284.8	1.08	65788.9	84.25
C1s B	286.02	1.08	6277.33	8.04
C1s C	287.14	1.09	1913.42	2.45
C1s D	288.49	1.08	740.27	0.95
O1s A	533.95	1.57	1572.28	0.72
O1s B	532.3	1.57	4091.95	1.87
O1s C	530.59	1.57	753.14	0.34
N1s A	398.74	1.8	814.81	0.63
N1s B	401.18	1.8	957.23	0.74

TABLE S10. XPS peak table for CNS annealed with H_3BO_3.

Name	Peak (eV)	FWHM (eV)	Area (CPS×eV)	At. %
C1s A	284.8	1.22	10689.16	8.82
C1s B	286.07	1.22	1665.75	1.37
C1s C	287.8	1.22	437.72	0.36
C1s D	287	1.22	773.61	0.64
C1s E	289.11	1.22	375.94	0.31
C1s F	290.54	1.22	292.74	0.24
O1s A	534.37	2.37	145629.47	42.95
O1s B	536.26	2.37	32724.06	9.65
B1s A	195.03	2.45	11000.32	23.93
B1s B	196.65	2.45	2977.1	6.48

TABLE S11. XPS peak table for CNS annealed with $Ca(OH))_2$.

Name	Peak (eV)	FWHM (eV)	Area (CPS×eV)	At. %
C1s A	284.8	1.22	27881.27	30.4
C1s B	286.51	1.83	5488.03	5.99
C1s C	288.42	1.83	2669.95	2.91
C1s D	291.1	1.83	12381.13	13.51
C1s E	293.42	1.83	1049.68	1.15
O1s A	532.89	1.77	75148.27	29.3
O1s B	534.19	1.77	13098.85	5.11
Ca2p3 A	348.65	1.83	38529.48	10.72
Ca2p3 B	353.49	0.8	595.83	0.17

[1] M. Karplus and G. K. Fraenkel, Theoretical Interpretation of Carbon-13 Hyperfine Interactions in Electron Spin Resonance Spectra, The Journal of Chemical Physics **35**, 1312 (1961).
[2] O. V. Yazyev, Hyperfine Interactions in Graphene and Related Carbon Nanostructures, Nano Letters **8**, 1011 (2008), arXiv:0702424 [cond-mat].
[3] R. C. Haddon and L. T. Scott, π-Orbital conjugation and rehybridization in bridged annulenes and deformed molecules in general: π-orbital axis vector analysis, Pure and Applied Chemistry **58**, 137 (1986).
[4] J. Sabalot-Cuzzubbo, G. Salvato-Vallverdu, D. Bégué, and J. Cresson, Relating the molecular topology and local geometry: Haddon's pyramidalization angle and the Gaussian curvature, The Journal of Chemical Physics **152**, 10.1063/5.0008368 (2020).
